# *sept7b* is required for the differentiation of pancreatic endocrine progenitors

**DOI:** 10.1038/srep24992

**Published:** 2016-04-26

**Authors:** Surjya Narayan Dash, Elina Hakonen, Jarkko Ustinov, Timo Otonkoski, Olov Andersson, Sanna Lehtonen

**Affiliations:** 1 Department of Pathology, University of Helsinki, Helsinki, Finland; 2 Research Program for Molecular Neurology and Biomedicum Stem Cell Center, University of Helsinki, Helsinki, Finland; 3 Department of Cell and Molecular Biology, Karolinska Institute, Stockholm, Sweden

## Abstract

Protection or restoration of pancreatic β-cell mass as a therapeutic treatment for type 1 diabetes requires understanding of the mechanisms that drive the specification and development of pancreatic endocrine cells. Septins are filamentous small GTPases that function in the regulation of cell division, cytoskeletal organization and membrane remodeling, and are involved in various tissue-specific developmental processes. However, their role in pancreatic endocrine cell differentiation remains unknown. Here we show by functional manipulation techniques in transgenic zebrafish lines that suppression of *sept7b*, the zebrafish ortholog of human *SEPT7*, profoundly increases the number of endocrine progenitors but limits their differentiation, leading to reduction in β- and α-cell mass. Furthermore, we discovered that *shh* (*sonic hedgehog*) expression in the endoderm, essential for the development of pancreatic progenitors of the dorsal pancreatic bud, is absent in larvae depleted of *sept7b*. We also discovered that *sept7b* is important for the differentiation of ventral pancreatic bud-derived cells: *sept7b-*depleted larvae exhibit downregulation of Notch receptors *notch1a* and *notch1b* and show precocious differentiation of *NeuroD*-positive endocrine cells in the intrapancreatic duct and gut epithelium. Collectively, this study provides a novel insight into the development of pancreatic endocrine progenitors, revealing an essential role for *sept7b* in endocrine progenitor differentiation.

Type 1 diabetes mellitus is characterized by a reduced number of functional β-cells, a feature that can also be seen in a subset of people with type 2 diabetes. Therefore one of the major objectives of diabetes research is to identify ways to restore the β-cell mass. This necessitates understanding of the basic mechanisms that underlie the formation of the pancreas and the specification of its cell types. During pancreatic development in mammals, endocrine and exocrine cells evolve from a common progenitor population^1^. The formation of early endocrine cells is often referred to as primary transition or the first wave, and the development of mature α, β, δ, ε and pancreatic polypeptide cells is referred to as secondary transition or the second wave of development^2^. Of the different endocrine cell types, the insulin-producing β-cells are the most prominent.

Development of β-cells encompasses coordinated functions of various conserved transcription factors in vertebrates. The homeodomain transcription factor Pdx1 is the initial pre-pancreatic endoderm marker^3^,^4^. Pdx1 is essential for the second wave of endocrine cell formation and remains highly expressed in most of the mature β-cells in mice^5^. Another transcription factor, the basic helix–loop–helix transcription factor Ptf1a, binds directly to the promoters of trypsin and elastase, specifying its role in exocrine cell differentiation^6^. During switching of progenitors to mature endocrine or exocrine cell types Pdx1 and Ptf1a function coordinately in the specification of multipotent progenitor cells^7^. However, despite continual advances in determining the molecular basis of pancreatic development, the genes that control the action of exocrine and endocrine factors involved in the specification and differentiation of pancreatic cell types is not well defined.

Septins, a family of GTP-binding proteins, were first identified in yeast in a screen for cell division mutants^8^. The expression of septins is conserved in vertebrates^9^, and they have been linked to a wide range of biological processes, including regulation of cell polarity^10^, exocytosis^11^,^12^,^13^ and mitosis^14^. Septin 7 localizes at the base of the primary cilium of epithelial cells and is required for ciliogenesis^10^,^15^,^16^,^17^ and as a diffusion barrier between the cilia and the apical plasma membrane^17^. We have previously shown that *sept7b*, the zebrafish ortholog of human septin 7, is essential for zebrafish kidney function and development of left-right asymmetry due to its role in ciliogenesis^15^. Septin 7 regulates dendritic branching and the morphology of dendritic spines during neuronal maturation^18^. It is also involved in meiosis in mouse oocytes^19^. Moreover, septin 7-deficient mouse embryos display defective cytokinesis and fail to gastrulate^20^, and as such the later functions of septin 7 *in vivo* are scarcely described. We showed previously that septin 7 regulates glucose transporter trafficking in the kidney glomerular epithelial cells^13^. However, it is not known whether septin 7 regulates glucose metabolism *in vivo* and plays a role in the development of pancreas.

We explored the specific requirement of *sept7b* in the formation of pancreas by manipulating *sept7b* gene expression and rescue experiments in zebrafish. Zebrafish has established itself as an excellent system to model human diseases^21^ and an attractive, transparent model to study mechanisms of pancreas formation^22^. It has also proven suitable for screening assays with an aim to identify small molecules that could be used to develop therapies for diabetes^23^. In zebrafish, pancreas development is characterized by spatially segregated endocrine and exocrine precursor populations, which fuse to form the pancreas^24^. Like mammals, the zebrafish possess two waves of endocrine cell development, and the development of late endocrine cells corresponds to differentiation of mature endocrine cells in mammals^25^,^26^,^27^. Also similar to mammals, early specification of endocrine progenitors in zebrafish is Pdx1 independent, but Pdx1 is essential for the formation of endocrine cells during the second wave^26^.

Several signalling cascades regulate the development of pancreas in zebrafish, including Hedgehog (Hh) and Notch pathways. Contrary to mammals, in which the increased activity of Hh inhibits the development of pancreas^28^, inhibition of Hh signalling at early stages of gastrulation in zebrafish leads to nearly complete absence of the endocrine pancreas indicating that Hh signalling is essential for the specification of endocrine cells^29^. The Notch-responsive cells in the ductal epithelium give rise to the endocrine cells that differentiate during secondary transition in the zebrafish larvae^25^,^30^. Loss of Notch signalling in zebrafish causes excessive differentiation of endocrine cells in the intrapancreatic duct, whereas ectopic activation of the Notch pathway inhibits both acinar cell differentiation and maintenance of β-cell differentiation in embryonic zebrafish^25^,^31^.

In this study, we elucidated the role of *sept7b* in the specification and differentiation of pancreatic progenitors during the development of pancreas using both transgenic reporter zebrafish models expressing pancreatic markers and wild type zebrafish. Whole-mount antibody staining and three-dimensional (3D) reconstruction of confocal z-stack images coupled with software-based quantification techniques was used to determine endocrine cell mass in the presence and absence of *sept7b*. We observed that knockdown of *sept7b* led to an upsurge in the accumulation of pancreatic progenitors in the islet, which, however, failed to differentiate into mature cell types, and thus reduced both insulin- and glucagon- positive cell mass. Collectively, the data suggest that *sept7b* is required for both early pancreas development, correlating with downregulation of Shh signalling during development of the dorsal pancreatic bud, and the secondary endocrine differentiation, correlating with downregulation of Notch signalling, which is important in ductal cells derived from the ventral pancreatic bud.

## Results

### Septin 7 expression is conserved in endocrine and exocrine pancreas in vertebrates

**Figure 1:**
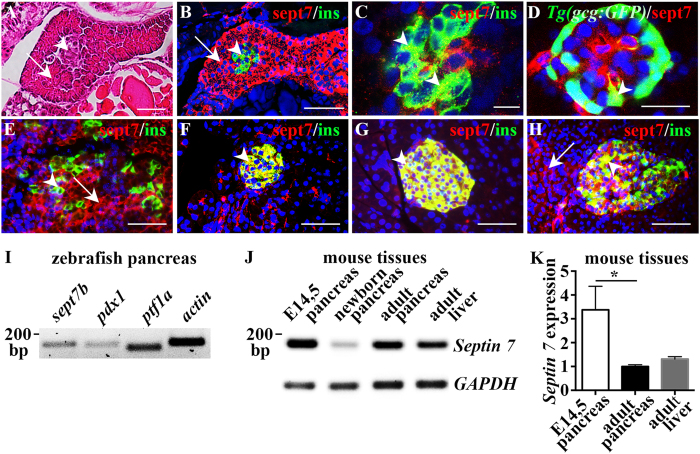
Expression of septin 7 in vertebrate pancreas. (**A**) Longitudinal section of 4 dpf zebrafish larva stained with hematoxylin and eosin shows endocrine (arrowhead) and exocrine cells (arrow). (**B**) Longitudinal section of 4 dpf zebrafish larva immunostained for septin 7 (red) and insulin (green) shows cytoplasmic localization of septin 7 in exocrine cells (arrow) and β-cells positive for insulin (arrowhead). (**C**) Confocal image of a 4 dpf zebrafish larvae immunostained as whole-mount for septin 7 (red) and insulin (green) confirms that septin 7 is expressed in β-cells (arrowheads). (**D**) Confocal image of 4 dpf *Tg(gcg:GFP*) zebrafish larva stained as whole mount shows localization of septin 7 (red) in glucagon-positive α-cells (arrowhead). (**E**) Septin 7 (red) shows cytoplasmic localization in E14.5 mouse pancreas in β-cells (arrowhead) stained for insulin (green) and in exocrine cells (arrow). (**F,G**) Septin 7 (red) concentrates in the islets (arrowheads) of newborn (**F**) and adult (**G**) mouse pancreas. Staining for insulin (green) visualizes β-cells. (**H**) In adult human pancreas septin 7 (red) localizes in the islets (arrowhead). Staining for insulin (green) visualizes β-cells. A few exocrine cells are also positive for septin 7 (arrow). In (**B–H**), nuclei are labeled with DAPI (blue). **(I)** RT-PCR confirms the expression of *sept7b* in adult zebrafish pancreas along with *pdx1, ptf1a,* and *actin.* (**J**) RT-PCR confirms the expression of *Septin 7* in embryonic day 14.5 (E14.5), newborn and adult pancreas and adult liver in mouse. *GAPDH* is used as a control. (**K**) Quantitative RT-PCR shows that the expression of *Septin 7* is 3-fold higher in embryonic pancreas than in the adult pancreas and liver. The difference in the expression level of *Septin 7* in the adult liver and adult pancreas is not significant. The expression of *Septin 7* was normalized to *Cyclophilin G*. Error bars represent mean ± SEM (n = 4–5). *p ≤ 0.05. Scale bar: (**A**) (25 μM), (**B**) (30 μM), (**C**) (7.5 μm), (**D**) (10 μm), (**E–H**) (30 μm).

To determine the physiological significance of septin 7 in the development of pancreas, we first analysed its expression in the pancreas of zebrafish larvae. Endocrine and exocrine compartments were visualised in the longitudinal section of a 4 days post fertilization (dpf) zebrafish larvae stained with haematoxylin-eosin (Fig. 1A). Double labelling of 4 dpf zebrafish larvae for insulin and septin 7 revealed that septin 7 is localized in the cytoplasm in both exocrine and endocrine cells (Fig. 1B). Whole mount immunostaining and confocal microscopy indicated that septin 7 localizes in insulin-positive β-cells (Fig. 1C). Labelling of *Tg*(*gcg:GFP*) reporter zebrafish larvae further revealed that septin 7 is also observed in α-cells positive for glucagon (Fig. 1D).

We found previously that the expression of septin 7 in the kidney is conserved in different vertebrates^15^. We therefore next studied whether septin 7 is also present in pancreas in higher vertebrates. Immunostaining revealed that in embryonic day 14.5 (E14.5) mouse pancreas, septin 7 localized to both endocrine and exocrine cells (Fig. 1E), whereas in newborn (Fig. 1F) and adult mouse pancreas (Fig. 1G) and adult human pancreas (Fig. 1H), septin 7 concentrated to islet cells and only few exocrine cells showed signal.

We further analysed the expression of *sept7b*, the zebrafish ortholog of human *SEPT7*, by conventional RT-PCR, and found that *sept7b* is expressed in the adult zebrafish pancreas together with transcription factors *pdx1* and *ptf1a* (Fig. 1I). Expression of *Septin 7* mRNA was also observed in E14.5, newborn and adult mouse pancreas, and in adult mouse liver by conventional RT-PCR (Fig. 1J). Quantitative RT-PCR revealed 3-fold higher level of *Septin 7* mRNA in E14.5 mouse pancreas compared to adult pancreas or liver (Fig. 1K). Collectively, these data confirm that the expression of septin 7 in pancreas is conserved across vertebrates.

### *sept7b* is crucial for β-cell development

Adequate β-cell mass is a necessity to produce enough insulin to maintain blood glucose homeostasis. To gain insight into the influence of *sept7b* in the development of pancreatic β-cells, we knocked down *sept7b* using a translation-blocking morpholino antisense oligonucleotide (TBMO) in zebrafish embryos. We first analysed the knockdown efficiency and specificity of the *sept7b* TBMO at 5 dpf. As previously shown by us, 88% of *sept7b* TBMO-injected larvae (n = 200) showed pericardial and yolk sac edema (Supplementary Fig. 1B) due to disturbed pronephric function^15^. Co-injection of *sept7b* TBMO and capped *sept7b* mRNA partially rescued the phenotype (Supplementary Fig. 1C), with only 14% of the larvae (n = 200) exhibiting the phenotype. Control MO-injected larvae (n = 150) showed no phenotypic changes (Supplementary Fig. 1A). Western blotting revealed that *sept7b* TBMO-injected larvae show 74% downregulation of septin 7 protein at 5 dpf (Supplementary Fig. 1D,F). We further tested whether *sept7b* TBMO causes general off-target effects by analysing, using qPCR, the expression level of *p21*, a downstream target of *p53*^32^. *p21* showed a non-significant trend of downregulation in *sept7b* TBMO-injected larvae (Supplementary Fig. 1E) further supporting the notion that *sept7b* TBMO does not cause off-target effects.

**Figure 2:**
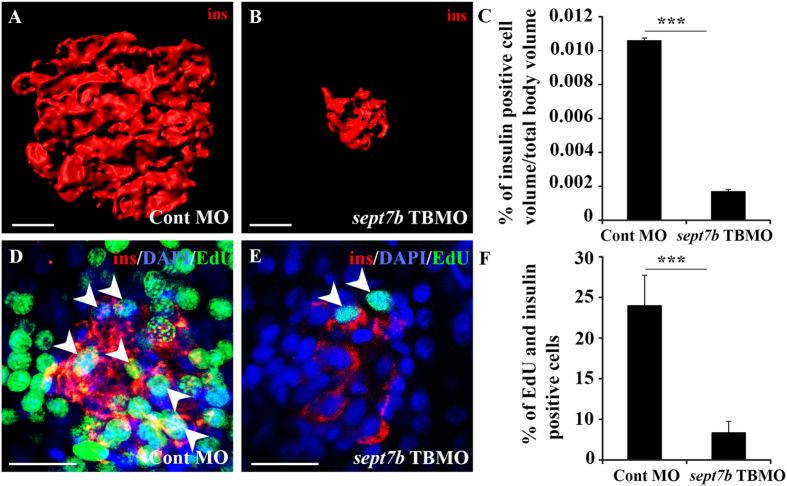
Knockdown of *sept7b* reduces β-cell volume and inhibits β-cell proliferation. (**A,B**) Three-dimensional rendered z-stack confocal images generated from control MO-injected (**A**) and *sept7b* TBMO-injected (**B**) zebrafish larva at 5 dpf stained as whole mounts with antibodies against insulin. (**C**) Insulin-positive cell volume is significantly reduced in *sept7b* TBMO-injected zebrafish larvae at 5 dpf. (**D,E**) Three-dimensional rendered z-stack confocal images generated from control MO-injected (**D**) and *sept7b* TBMO-injected (**E**) zebrafish larva at 5 dpf treated with EdU (green) to visualize proliferating cells and stained with antibodies against insulin (red) to visualize β-cells. DAPI shows the nuclei (blue). Arrowheads indicate proliferating β-cells (positive for both EdU and insulin). (**F**) β-cell proliferation is significantly reduced in *sept7b* knockdown larvae. Error bars represent mean ± SEM. Scale bar (**A,B**) (10 μm); (**D,E**) (10 μm). ***p ≤ 0.0005.

To define whether *sept7b* regulates the development of pancreatic β-cells, we stained control MO- and *sept7b* TBMO-injected larvae at 5 dpf as whole-mounts with an antibody against insulin (Fig. 2A,B). We then created the isosurface of the insulin-positive β-cells by 3D rendering of the confocal images, and quantified the surface volume of β-cells relative to total body volume. We found that insulin-positive β-cell volume was reduced by 84% in *sept7b* TBMO-injected larvae compared to control MO-injected larvae (Fig. 2C). To analyse whether the reduced insulin-positive cell volume is due to defects in β-cell proliferation, we stained the control MO and *sept7b* TBMO-injected 5 dpf larvae as whole mounts with an antibody against insulin, and analysed proliferation by incorporation of EdU into the DNA (Fig. 2D,E). Calculation of cells positive for both insulin and EdU in 3D reconstructed z-stack images indicated 80% reduction in β-cell proliferation rate in *sept7b* knockdown larvae compared to control MO-injected larvae (Fig. 2F). The results indicate that *sept7b* is crucial for the development of a normal β-cell mass, as well as for β-cell proliferation, which contributes to the expansion of newly differentiated β-cells.

### Downregulation of *sept7b* results in reduced α-cell volume and increased numbers of insulin and glucagon double positive cells

**Figure 3:**
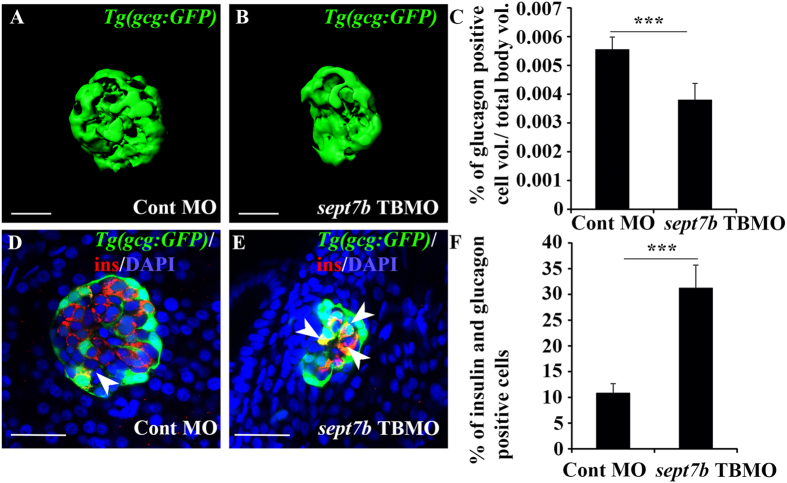
Knockdown of *sept7b* reduces α-cell volume and increases bihormonal cells positive for both insulin and glucagon. (**A,B**) Isosurface rendered z-stack confocal images generated from control MO-injected (**A**) and *sept7b* TBMO-injected (**B**) *Tg(gcg:GFP*) zebrafish larva at 4 dpf. Glucagon-positive α-cells are green. (**C**) Glucagon-positive cell volume is significantly reduced in *sept7b* TBMO-injected zebrafish larvae at 4 dpf. (**D,E**) Cells positive for both insulin (red) and glucagon (green) (bihormonal cells; arrowheads) are increased in *sept7b* TBMO-injected *Tg(gcg:GFP*) zebrafish larvae 4 dpf (**E**) compared to control MO-injected larva (**D**). In **(D**,**E**), nuclei are labeled with DAPI (blue). (**F**) Quantification of the percentage of bihormonal cells relative to insulin positive cells. Scale bar: (**A,B**) (25 μm); (**D,E**) (20 μm). Error bars represent mean ± SEM. ***p ≤ 0.0005.

The findings that septin 7 is expressed in the glucagon-positive α-cells (Fig. 1D) and knockdown of *sept7b* leads to a reduction in the β-cells mass (Fig. 2A–C), led us to investigate whether *sept7b* knockdown also affects α-cell development. To do this, we injected *sept7b* TBMO into *Tg*(*gcg:GFP*) zebrafish larva. 3D-reconstruction of the z-stack images and measurement of surface volume relative to the total body volume revealed that glucagon-positive α-cell volume was reduced by 32% in *sept7b* knockdown larvae compared to control MO-treated larvae at 4 dpf (Fig. 3A–C). We further quantified whether the number of bihormonal cells, expressing both insulin and glucagon, is increased by immunostaining control MO- and *sept7b* TBMO-injected *Tg*(*gcg:GFP*) zebrafish larvae with antibodies against insulin at 4 dpf. We found 3-fold higher level of cells positive for both glucagon and insulin in *sept7b* knockdown larvae compared to controls (Fig. 3D–F). This further demonstrates a failure in endocrine differentiation and suggests that *sept7b* might instigate the inter-endocrine cellular conversion.

### Knockdown of *sept7b* leads to inadequate development of the exocrine pancreas

**Figure 4:**
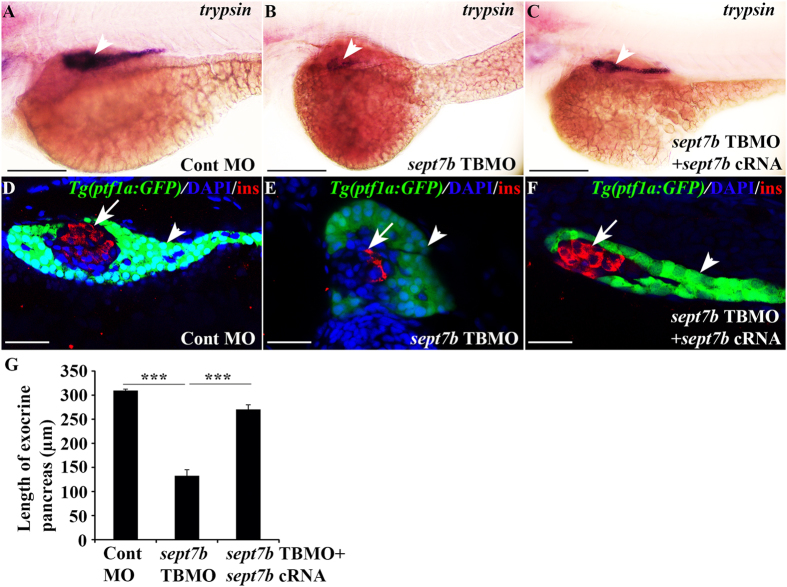
Depletion of *sept7b* leads to downregulation of exocrine marker *trypsin* and abnormal exocrine morphology. (**A,B**) Expression *of trypsin* in exocrine pancreas (arrowhead) is reduced in 3 dpf *sept7b* knockdown larvae (**B**) compared to control MO-injected larvae (**A**). (**C**) Co-injection of *sept7b* cRNA with *sept7b* TBMO restores *trypsin* expression (arrowhead). (**D,E**) *sept7b* TBMO-injected *Tg(ptf1a:GFP)* zebrafish larvae (**E**) show abnormal exocrine morphology and reduced size of the pancreatic tail (arrowhead) compared to the control MO-injected larvae (**D**) at 3 dpf. β-cells are labeled with insulin (red; arrow). (**F**) Co-injection of *sept7b* cRNA with *sept7b* TBMO partially restores the morphology of exocrine pancreas. In (**D–F**), nuclei are labeled with DAPI (blue). (**G**) The length of the exocrine pancreas, measured from 3 dpf zebrafish embryos using *trypsin* as a marker, is significantly decreased by *sept7b* knockdown compared to the control. Co-injection of *sept7b* cRNA with *sept7b* TBMO restores the length of the exocrine pancreas. Scale bar: (**A–C**) (75 μm); (**D–F**) (25 μm). Error bars represent mean ± SEM. ***p ≤ 0.0005.

To study whether *sept7b* plays a role in the development of exocrine pancreas, we knocked down *sept7b* and performed *in situ* hybridization for *trypsin*, a digestive enzyme expressed in the differentiated pancreatic exocrine cells^33^. The mRNA expression of *trypsin* at 3 dpf was markedly reduced in 85% of *sept7b* knockdown embryos (n = 60) compared to controls (n = 50) (Fig. 4A,B). To rescue the defect associated with knockdown of *sept7b*, we co-injected capped *sept7b* mRNA with *sept7b* TBMO, which restored the expression of *trypsin* in 84% of the *sept7b* knockdown embryos (n = 45) (Fig. 4C). We also knocked down *sept7b* in *Tg(ptf1a:GFP)* zebrafish embryos. The basic helix–loop–helix transcription factor Ptf1a is the pancreas-specific subunit of the heterotrimeric pancreas transcription factor 1 complex (PTF1), and is required for the differentiation of exocrine pancreas^34^. 80% of *Tg(ptf1a:GFP)* zebrafish larvae treated with *sept7b* TBMO (n = 15) showed reduced size and abnormal morphology of the exocrine pancreas at 3 dpf compared to control MO-injected larvae, which did not display any phenotypic changes (n = 15) (Fig. 4D,E). 73% of the larvae co-injected with *sept7b* capped mRNA and *sept7b* TBMO (n = 15) showed restored size and morphology of the exocrine pancreas (Fig. 4F). Quantification using *trypsin* expression as a marker revealed that the length of exocrine pancreas reduced from 309.45 ± 3.07 μm in the control MO-injected larvae to 132.61 ± 12.64 μm in the *sept7b* knockdown larvae (n = 15 each) (Fig. 4G). The length of the exocrine pancreas was significantly increased in the rescued larvae (270.39 ± 9.59 μm; n = 15) compared to the *sept7b* knockdown larvae (Fig. 4G). These results show that *sept7b* is required for the proper formation of exocrine pancreas.

### Progenitors positive for Pdx1 are increased in *sept7b* knockdown larvae

**Figure 5:**
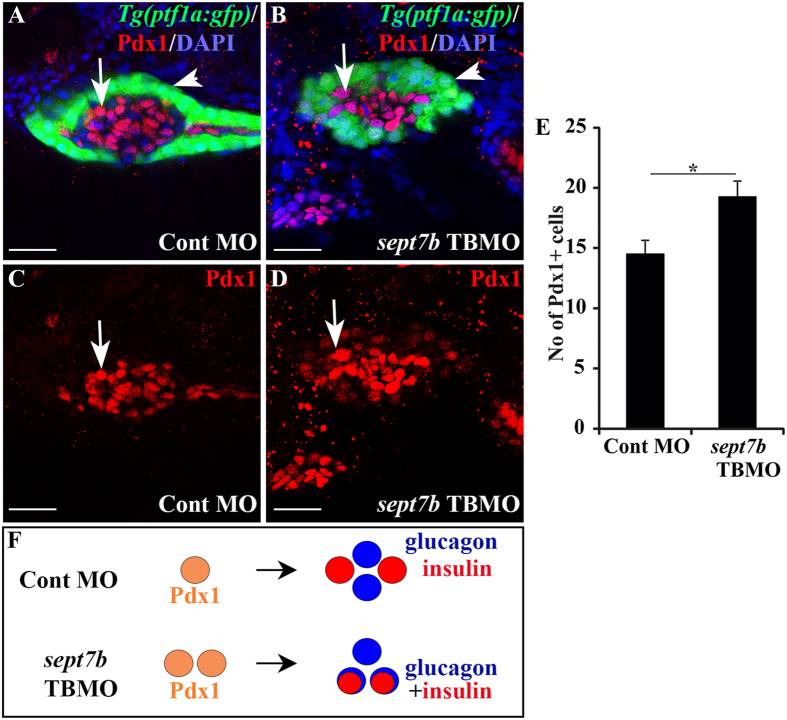
Pancreatic progenitors are increased in *sept7b* knockdown larvae. (**A–D**) Pdx1-positive cells (red; arrow) in control MO-injected (**A,C**) and *sept7b* TBMO-injected (**B,D**) *Tg(ptf1a:GFP*) zebrafish larvae at 3 dpf. In (**A**,**B**) the exocrine pancreas (arrowhead) is visualized by *ptf1a* (green) and the nuclei are labelled with DAPI (blue). (**C**) and (**D**) are corresponding images visualizing Pdx1 only. (**E**) Pdx1-positive cells are significantly increased in *sept7b* knockdown larvae compared to control MO-injected larvae. (**F**) Schematic representation showing that control MO-injected larva show differentiation of Pdx1-positive pre-pancreatic progenitors, which give rise to mature insulin- and glucagon-positive β- and α-cells, respectively. The islet cells in *sept7b* TBMO-injected larva fail to differentiate and present bihormonal cells positive for both insulin and glucagon (see also Fig. 3). Scale bar: (**A–D**) (25 μm). Error bars represent mean ± SEM. *p ≤ 0.05.

As our results showed that both endocrine and exocrine differentiation is disturbed in *sept7b* knockdown larvae, we next asked whether *sept7b* affects pancreatic progenitors, with a focus on the endocrine compartment. Reduced mass of β- and α- cells could be caused either by reduction in the number of endocrine progenitors or by a defect in their differentiation. To address this, we assessed whether reduced level of *sept7b* affects the number of progenitors expressing transcription factor Pdx1, which is required for pancreatic development^7^. We immunostained *sept7b* TBMO (n = 12) and control MO-treated (n = 12) *Tg*(*ptf1a:GFP*) zebrafish at 3 dpf with antibodies against Pdx1 (Fig. 5A–D), and found 32% more cells positive for Pdx1 in *sept7b* morphants than in control MO-injected embryos (Fig. 5E), further suggesting that a larger population of progenitor cells remained undifferentiated when *sept7b* was knocked down. We next performed a similar analysis at 5 dpf, revealing a persistent increase (21%) in the number of Pdx1-positive cells (Supplementary Fig. S2). Collectively, the schematic cartoon in Fig. 5F illustrates Pdx1-positive pancreatic progenitors that differentiate into mature endocrine cells, positive either for insulin or glucagon, in control MO-injected larvae. In the absence of *sept7b*, the numbers of Pdx-positive progenitors as well as cells double positive for insulin and glucagon increase (Fig. 5F), indicating that specification of endocrine cell types fails in the absence of *sept7b*.

### shh is downregulated in the endoderm in *sept7b* knockdown embryos

**Figure 6:**
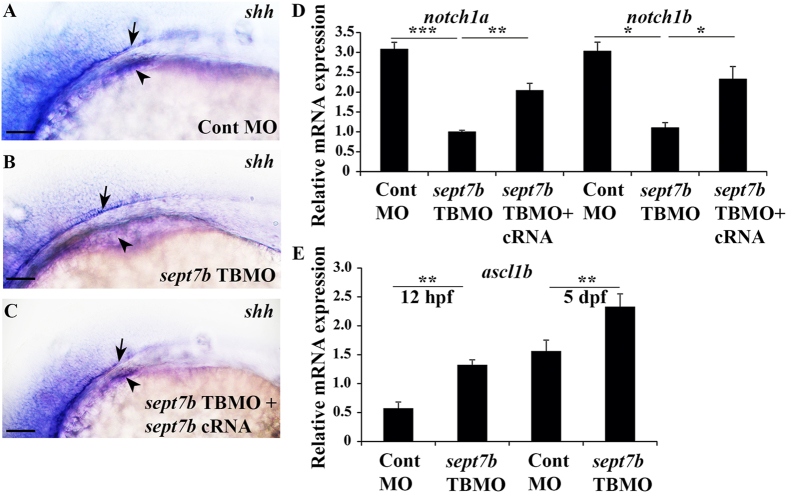
*Shh* expression is missing from endoderm of *sept7b* knockdown embryos. (**A**) Lateral view of 24 hpf zebrafish embryo injected with the control MO showing expression of *sonic hedgehog* (*shh)* in the endoderm (arrowhead) and notochord (arrow). (**B**) Endodermal expression of *shh* (arrowhead) is missing from *sept7b* knockdown embryos whereas expression in notochord is present (arrow). (**C**) Co-injection of *sept7b* cRNA with *sept7b* TBMO partially rescues the expression of *shh* in the endoderm (arrowhead). (**D**) *Notch* receptor expression is downregulated in *sept7b* knockdown larvae. The expression of *notch1a* and *notch1b* mRNAs is significantly downregulated in *sept7b* knockdown larvae compared to control MO-injected larvae. Co-injection of *sept7b* cRNA with *sept7b* TBMO partially rescues downregulation of *notch1a* and *notch1b*. The expression of *notch1a* and *notch1b* were normalized to actin. (**E**) The expression of *ascl1b* mRNA is increased at 12 hpf and 5 dpf in *sept7b* TBMO-injected larvae compared to control MO-injected larvae. Scale bar: (**A–C**) (50 μm). Error bars represent mean ± SEM. *p ≤ 0.05; **p ≤ 0.005; ***p ≤ 0.0005.

We next delineated the signalling pathways that *sept7b* regulates to mediate both endocrine and exocrine differentiation. The Hedgehog (Hh) signalling pathway contributes to cell differentiation and organ development, and its activity is required for the specification of pancreas and differentiation of endocrine cells in zebrafish^29^,^35^. We performed *in situ* hybridization to detect *sonic hedgehog* (*shh*) expression in 24 hpf zebrafish embryos injected with control MO and *sept7b* TBMO. 68% of *sept7b* knockdown embryos (n = 70) displayed *shh* expression in the notochord but no signal in the endoderm whereas 92% of control MO-injected embryos showed signal in both the notochord and endoderm (n = 70) (Fig. 6A,B). Co-injection of *sept7b* capped mRNA and *sept7b TBMO* re-established the expression of *shh* in the endoderm in 80% of the embryos (n = 50) (Fig. 6C). Our data suggest that the absence of endodermal expression of *shh* after *sept7b* knockdown may underlie the improper development of dorsal bud-derived endocrine pancreas.

### *sept7b* knockdown downregulates Notch signalling and increases cells positive for NeuroD in principal islets

The Notch pathway regulates endocrine progenitor differentiation during the formation of both primary and secondary islets in zebrafish, and interestingly, different levels of Notch activity define the fates of the progenitors^36^. To determine whether *sept7b* regulates the activity of the Notch pathway, we first quantified the mRNA expression levels of zebrafish Notch receptors, *notch1a* and *notch1b* (orthologs of human *NOTCH*), as a measure of Notch activation. Both *notch1a* and *notch1b* were significantly downregulated in *sept7b*-depleted larvae at 5 dpf, and this was partially rescued by co-injection of capped *sept7b* mRNA with *sept7b* TBMO (Fig. 6D). This indicates that depletion of *sept7b* suppresses the activation of the Notch pathway by downregulating the *notch1* receptors in zebrafish larvae.

**Figure 7:**
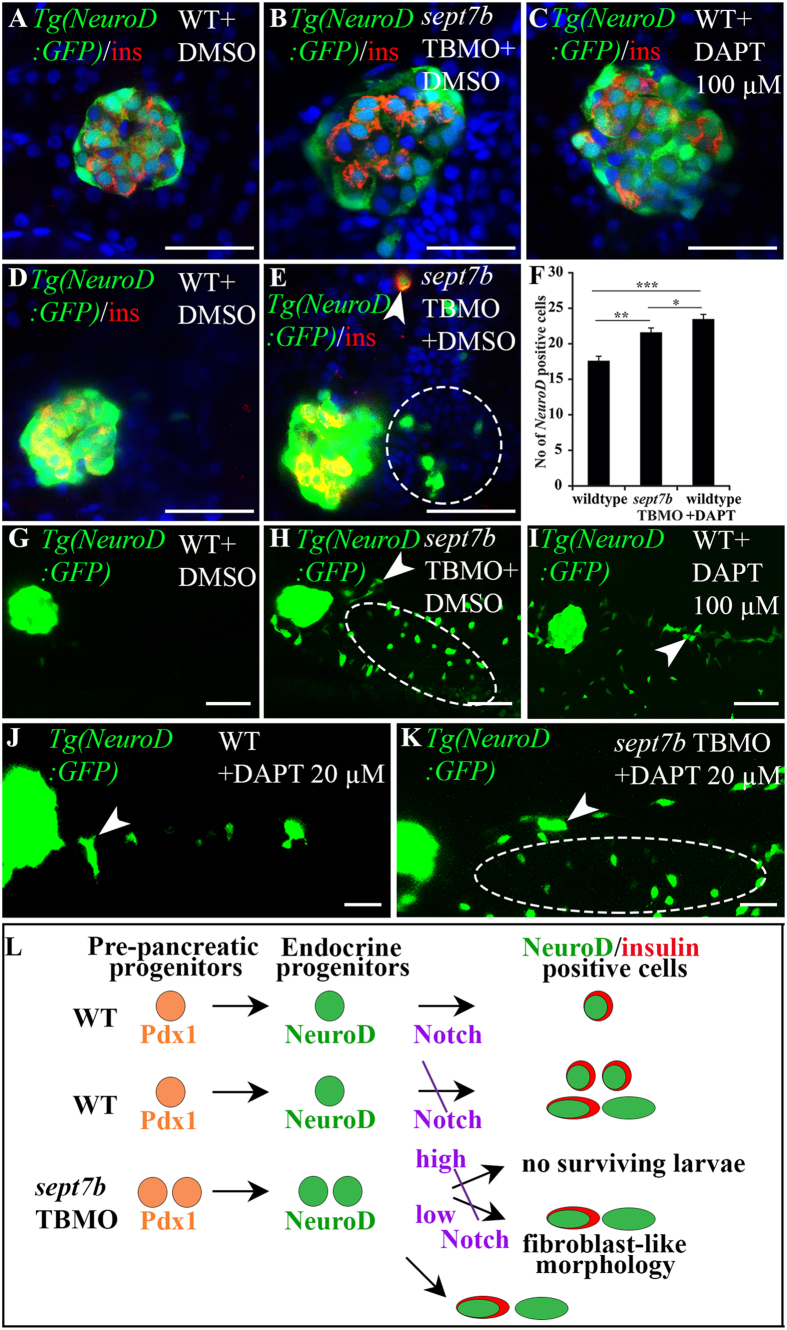
Endocrine progenitors are increased in islets, exocrine pancreas and gut epithelium in *sept7b*-depleted larvae. (**A–C**) 5 dpf *Tg(NeuroD:GFP*) zebrafish larva treated with DMSO **(A)**, *sept7b* TBMO (**B**), or 100 μM Notch-inhibitor DAPT **(C)** stained for insulin (red). (**D,E**) Enhanced exposure of 5 dpf *Tg(NeuroD:GFP*) larvae treated with DMSO (**D**) or *sept7b* TBMO (**E**) reveal excessive induction of *NeuroD*-positive cells in the extrapancreatic duct of *sept7b*-depleted larvae (circle). Occasional insulin and *NeuroD* double*-*positive cells (arrowhead) are observed in the intrapancreatic duct (IPD) of *sept7b-*depleted larva but not in controls. (**F**) *NeuroD*-positive endocrine cells are increased in *sept7b* TBMO- and 100 μM DAPT-treated larvae compared to DMSO-treated controls. (**G–I**) Enhanced exposures of 5 dpf *Tg(NeuroD:GFP*) larva treated with DMSO (**G**), *sept7b* TBMO (**H**), or 100 μM DAPT (**I**) reveal *NeuroD*-positive cells in exocrine pancreas (arrowhead) and gut epithelium (ellipse) in *sept7b* TBMO- and DAPT-treated larvae (**H,I**) but not in controls (**G**). (**J,K**) 20 μM DAPT causes *NeuroD*-positive endocrine cell formation (arrowhead) in the IPD and gut epithelium (ellipse) in *sept7b*-depleted larvae (**K**). Only few *NeuroD*-positive cells (arrowhead) are observed in the IPD of 20 μM DAPT-treated controls (**J**). (**L**) Schematic representation of pancreatic progenitor differentiation in wild type (WT), *sept7b* TBMO-treated and Notch-inhibited zebrafish larvae. In WT larva early Pdx1-positive pre-pancreatic progenitors give rise to late *NeuroD*-positive endocrine progenitors. Notch-responsive endocrine progenitors differentiate into *NeuroD*- and insulin-positive β-cells. Inhibition of Notch by 100 μM DAPT in WT larvae leads to differentiation of numerous *NeuroD* and insulin-positive progenitors in the islets. Notch inhibition also leads to emergence of *NeuroD*-positive cells in the pancreatic tail region, with fibroblast-like morphology. *sept7b* depletion leads to similar phenotype as inhibition of Notch: *NeuroD*-positive progenitors increase in the endocrine and exocrine pancreas, with fibroblast-type morphology. Treatment of *sept7b*-depleted larvae with 100 μM DAPT (high) leads to lethal toxicity, whereas 20 μM DAPT (low) is less toxic and the surviving larvae show induction of endocrine cells in the IPD and gut epithelium. Error bars represent mean ± SEM. *p ≤ 0.05; **p ≤ 0.01; ***p ≤ 0.005. Scale bar: (**A–E**) (25 μm); (**G–I**) (50 μm); (**J–K**) (30 μm).

Basic helix-loop-helix transcription factors, Ascl1b and Neurod1 control the endocrine cell fate and are downregulated by Notch signalling^37^,^38^. Quantitative PCR analysis revealed an increase in *ascl1b* at both 12 hpf and 5 dpf in *sept7b* TBMO-treated embryos compared to control MO-treated ones (Fig. 6E). This suggests that upon downregulation of Notch receptors, *ascl1b* is upregulated in *sept7b* knockdown larvae. We next knocked down *sept7b* in the *Tg(NeuroD:GFP)* reporter zebrafish line, and found that *NeuroD*-positive cells increased by 19% and 18% in 3 and 5 dpf *sept7b* knockdown larvae compared to controls (n = 15/group in both time points) (Supplementary Fig. S3, Fig. 7A,B,F). These data show that depletion of *sept7b* increases the numbers of endocrine progenitors in the principal islets.

### Differentiation of Notch-responsive secondary endocrine progenitors requires *sept7b*

Notch-responsive cells in the pancreatic ductal epithelium in larval zebrafish represent a population of progenitor cells that act as a source of secondary islets, and inhibition of the Notch pathway leads to precocious differentiation of endocrine cells in the ductal region^25^. Interestingly, we observed *NeuroD*-positive cells outside of the principal islet, in the intrapancreatic duct (IPD), pancreatic tail region and gut epithelium in *sept7b* knockdown larvae (Fig. 7E,H), but not in the controls (Fig. 7D,G). Furthermore, labelling of *sept7b*-depleted *Tg(NeuroD:GFP)* zebrafish larvae for insulin revealed occasionally cells double positive for insulin and *NeuroD* in the IPD and gut epithelium (Fig. 7E). Thus the excessive induction of endocrine cells in the ductal and gut epithelium after *sept7b* knockdown resembles the pancreatic phenotype observed when Notch signalling is inhibited in zebrafish^25^,^26^,^36^. To further define whether Notch-responsive endocrine cell differentiation during secondary islet formation requires *sept7b* and whether the precocious induction of *NeuroD*-positive endocrine cells in the IPD and gut epithelium is due to deregulation of the Notch-pathway after depletion of *sept7b*, we combined knockdown of *sept7b* with inhibition of Notch. Inhibition of Notch by 100 μM DAPT in wild type embryos (n = 15) (Fig. 7C) led to 25% increase in the number of *NeuroD*-positive endocrine progenitors in the principal islets compared to the wild type untreated larvae (n = 15) (Fig. 7F). We also observed induction of endocrine cell formation in the IPD and pancreatic tail regions in the Notch-inhibited wild type larvae (Fig. 7I) similarly as after *sept7b* knockdown (Fig. 7E,H). Treatment of *sept7b* knockdown larvae with 100 μM DAPT led to 96% mortality of the larvae (n = 150), whereas treatment with 20 μM DAPT led to only 62% mortality (n = 85), and the surviving larvae exhibited *NeuroD*-positive endocrine cell induction in the IPD and pancreatic tail region as well as in the gut epithelium (Fig. 7K). On the contrary, only few cells positive for *NeuroD* were observed in the IPD and pancreatic tail region, but not in the gut epithelium of the control larvae treated with 20 μm DAPT (Fig. 7J). We further found occasional (zero to two per larva) insulin positive cells in the exocrine pancreas of the wild type and *sept7b* knockdown larvae treated with 20 μM DAPT. However, insulin-positive cells increased 3-fold in the *sept7b* knockdown larvae treated with 20 μM DAPT compared to the wild type larvae treated with 20 μM DAPT (Supplementary Fig. S4). This suggests that Notch-responsive cells that give rise to secondary islets differentiate in the ductal and gut epithelium after *sept7b* depletion. Collectively, our data indicate a role for *sept7b* in the differentiation of the Notch-responsive endocrine progenitors in the zebrafish larvae as visualised in the schematic cartoon in Fig. 7L. These data correlate with a scenario where depletion of *sept7b* inhibits Notch activity in the pancreas in zebrafish larvae and leads to induction of endocrine cell formation in the ductal epithelium, similar to inhibition of Notch in the wild type larvae. The additive effect of *sept7b* knockdown coupled with treatment with a low concentration of Notch inhibitor further suggests that *sept7b* regulates the Notch pathway (Fig. 7L).

### *Sept7b* is essential for maintaining glucose homeostasis

**Figure 8:**
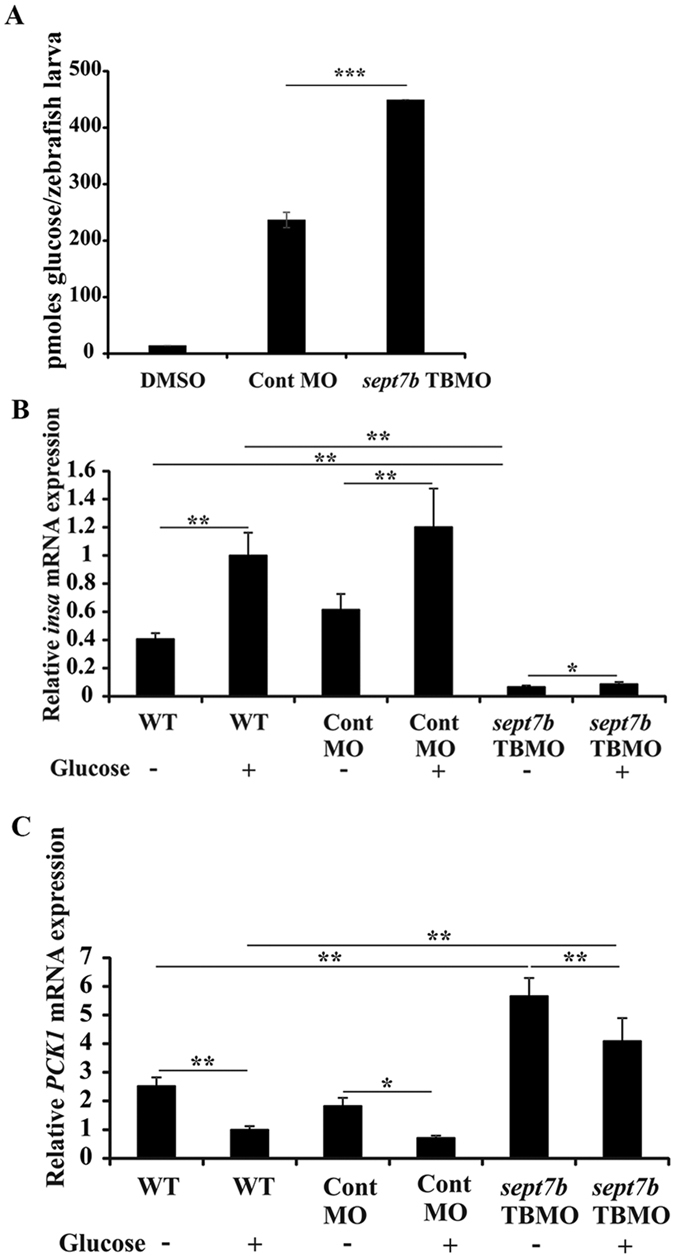
Knockdown of *sept7b* affects glucose homeostasis. (**A**) The amount of whole body glucose is significantly increased in *sept7b* knockdown larva compared to control MO-injected larvae at 5 dpf. DMSO in buffer served as a negative control. (**B**) qRT-PCR indicates that 40 mM exogenous glucose treatment for 24 h increases *insa* expression in wild type and control MO-injected zebrafish larvae at 5 dpf. *insa* expression is downregulated in *sept7b* knockdown larvae compared to the controls. Treatment of *sept7b* knockdown larvae with exogenous glucose increases *insa* expression, but the level remains clearly lower compared to wild type and control MO-treated larvae with or without exogenous glucose treatment. (**C**) qRT-PCR indicates that 40 mM exogenous glucose treatment for 24 h downregulates *pck1* expression in wild type and control MO-injected zebrafish larvae at 5 dpf. *pck1* expression is upregulated in *sept7b* knockdown larvae compared to the controls. Treatment of *sept7b* knockdown larvae with exogenous glucose decreases *pck1* expression, but the level remains clearly higher compared to wild type and control MO-treated larvae with or without exogenous glucose treatment. This indicates defective regulation of glucose homeostasis in *sept7b* knockdown larvae. In (**B**,**C)**, *insa* and *pck1* were normalized to β-actin. Error bars represent mean ± SEM. *p ≤ 0.05; **p ≤ 0.005; ***p ≤ 0.0005.

The failure of pancreatic differentiation could be expected to lead to alterations in the glucose level in *sept7b* knockdown larvae. We therefore measured the total amount of glucose in *sept7b* TBMO- and control MO-treated zebrafish larvae at 5 dpf. Knockdown of *sept7b* increased the absolute glucose level of the larvae by 90% compared to the controls (Fig. 8A). We next asked whether the increase in whole body glucose is due to reduced production of insulin, as could be expected based on the reduced β-cell mass in *sept7b* morphants, and whether there are changes in hepatic glucose production. We treated 4 dpf wildtype, control MO- and *sept7b* TBMO-treated zebrafish with exogenous glucose for 24 h, and measured the expression of *insa* (insulin a gene) by qRT-PCR. Larval zebrafish are expected to absorb exogenous glucose and, in response, to increase *insa* expression^39^. As expected, in both wild type and control MO-treated larvae, glucose increased *insa* expression 1.5-fold (Fig. 8B). In *sept7b* knockdown larvae *insa* expression was significantly lower compared to wild type and control MO-treated larvae (Fig. 8B). Glucose increased *insa* expression in *sept7b* morphants, but only by 25%, and the level remained clearly lower than in the controls (Fig. 8B).

We next assessed the expression of phosphoenolpyruvate carboxykinase 1 (*pck1*), which catalyses the rate-limiting step in gluconeogenesis in zebrafish similarly as in mammals^39^. Expression of *pck1* is induced in fasting conditions increasing hepatic glucose production, and insulin that is secreted after feeding, suppresses *pck1*. We observed that in both wild type and control MO-treated zebrafish larvae, administration of exogenous glucose reduced *pck1* expression as expected (Fig. 8C). In *sept7b* knockdown larvae *pck1* expression was significantly higher compared to wild type and control MO-treated larvae (Fig. 8C). Glucose decreased *pck1* expression in *sept7b* morphants, but the level remained clearly higher than in the controls (Fig. 8C). The results indicate that knockdown of *sept7b* leads to reduced expression of *insa*, enhanced expression of *pck1*, and thereby elevation of whole body glucose level.

## Discussion

This study provides evidence that the small GTPase *sept7b* regulates β-cell proliferation and plays an essential role in the differentiation of endocrine and exocrine pancreas in zebrafish. Specifically, we found that progenitors intended to give rise to mature endocrine cells accumulated and failed to differentiate upon depletion of *sept7b* leading to reduced β- and α-cell mass and impaired glucose homeostasis.

Our study shows that *sept7b* is important for β-cell proliferation. This is supported by previous studies showing that septin 7 is essential for cytokinesis in budding yeast and mammalian cells in a cell-type specific manner^20^. Furthermore, mice depleted of *Sept7* show early embryonic lethality apparently due to defective mitosis^20^. Our finding that β-cells proliferate in wild type larvae is supported by a previous study, which revealed that self-replication of β-cells is important in the generation of new β-cells in larval zebrafish^40^. Lack of *sept7b* could thus lead to reduced β-cell replication, contributing to reduced β-cell mass. However, other mechanisms, such as neogenesis of β-cells, may also be distorted and contribute to the reduction in β-cell mass following knockdown of *sept7b.*

Interestingly, our study also revealed reduction of glucagon-positive α-cell mass in addition to the reduced β-cell mass, and an increase in the number of bihormonal cells positive for both glucagon and insulin upon depletion of *sept7b*. This suggests that differentiation of pancreatic progenitors is disturbed in the absence of *sept7b*. In zebrafish, insulin-expressing cells are detected before glucagon positive cells appear, and by 24 hpf both hormones can be detected in the presumptive islet^33^. Insulin and glucagon double-positive cells have been observed in both human and rodents at early developmental stages^41^,^42^. Lineage tracing studies in mouse indicated that adult insulin- and glucagon-producing cells differentiate from two independent cell lineages, which apparently arise from a common precursor during morphogenesis^43^. Earlier studies in zebrafish failed to detect insulin and glucagon double-positive cells in wild type zebrafish^33^,^40^, but a recent report using insulin and glucagon reporter zebrafish lines identified double-positive cells also in wild type larvae^44^. The same study also describes transdifferentiation of α-cells to β-cells after severe destruction of β-cells in larval zebrafish, and thus appearance of cells double-positive for insulin and glucagon^44^. The finding of insulin and glucagon double-positive cells in *sept7b* knockdown larvae suggests that *sept7b* regulates the specification of endocrine cell fate at an early pancreatic precursor stage. We cannot, though, rule out the possibility that reduced β-cell mass after depletion of *sept7b* induces transdifferentiation of α-cells to β-cells.

Transcription factors Pdx1 and NeuroD are central for pancreatic cell differentiation, and interestingly, we observed increased numbers of cells positive for Pdx1 and *NeuroD* after *sept7b* depletion. Pdx1 is an early marker of pancreatic differentiation and appears in pancreatic endoderm progenitors before insulin^3^,^4^. Pdx1-expressing progenitors then give rise to both exocrine and endocrine lineages^43^. In zebrafish, the formation of late endocrine cells during the second wave of development, contrary to the first wave, requires Pdx1^26^. Also NeuroD is an early marker of endocrine precursors and its expression persists in mature endocrine cells^37^,^45^. Differential levels of NeuroD regulate endocrine cell fate decision^46^. Increased numbers of cells positive for Pdx1 and *NeuroD* together with reduced β- and α-cell mass in *sept7b*-depleted larvae indicates the necessity of *sept7b* for the differentiation of endocrine progenitors to mature hormone-producing cells.

The Hh signalling pathway plays a central role in the development of pancreas. Our study revealed that *shh* is missing from the endoderm in *sept7b* knockdown embryos whereas its expression remains in the notochord. The role of Hh signalling in the development of pancreas differs in mammals and zebrafish. In mammals, increased Hh signalling blocks the morphogenesis of pancreas, whereas in zebrafish, lack of Hh signalling leads to defective endocrine cell specification and absence of pancreatic endocrine markers^28^,^29^,^35^. The Shh receptor *smo* mutant zebrafish lacks dorsal pancreatic β-cells, whereas the later developing ventral pancreatic bud-derived β-cells do develop^47^. Interestingly, cell transplantation experiments revealed that the endodermal cells adjacent to the β-cell precursors required the function of *smo* for the induction of β-cells to occur, and that intra-endodermal interactions were essential for the process^47^. In our study, depletion of *sept7b* led to lack of *shh* in endoderm apparently leading to disturbed induction of β-cells in the dorsal pancreatic bud. The role of septins in regulating Shh signalling is supported by a previous study revealing that depletion of septin 2 reduced accumulation of Smo in cilia, thereby disturbing Shh signalling in this organelle^17^.

The Notch signalling pathway controls pancreatic cell differentiation by maintaining a population of precursor cells in the pancreatic ductal epithelium in an undifferentiated state and thus provides a source for secondary endocrine cells that will ultimately produce the secondary islets^25^,^26^,^36^. The mechanisms by which Notch signalling regulates pancreatic endocrine progenitors is complex, however, as different levels of Notch signalling lead to a different outcome, high levels inducing quiescence and sustained low levels promoting progenitor amplification followed by differentiation of endocrine cells^36^. We found that knockdown of *sept7b* reduced the expression levels of zebrafish Notch receptors *notch1a* and *notch1b*. Furthermore, similar to the state when Notch signalling is inhibited^25^,^36^, we found excessive induction of *NeuroD*-positive endocrine cells in the intrapancreatic duct, pancreatic tail region and gut epithelium, and also occasionally observed cells double positive for insulin and *NeuroD*. We also observed upregulation of *ascl1b*, a transcription factor required for the generation of the first pancreatic precursors^37^, in the absence of *sept7b*. As Notch signalling suppresses the expression of *NeuroD* and *ascl1b*, the increase in *NeuroD* and *ascl1b* upon knockdown of *sept7b* supports the idea that Notch signalling is inhibited in the absence of *sept7b*, corroborating a link between *sept7b* and the second wave of endocrine differentiation. Similarly, *mib* (Mind bomb, Delta ubiquitin ligase) mutation in zebrafish, which leads to failure in Notch signalling mediated via Notch ligand Delta, leads to markedly elevated *NeuroD* expression in the gut epithelium^45^. Interestingly, *mib* mutants and wild type embryos treated with Notch inhibitor DAPT show reduced numbers of α-cells^45^, similar to what we observed in *sept7b* knockdown larvae. Furthermore, *sept7b* knockdown larvae, similar to *mib* mutants, show reduced numbers of cells positive for the exocrine marker trypsin^45^. Collectively, the data support a role for *sept7b* in the differentiation of endocrine and exocrine pancreas involving modulation of Notch signalling by increasing the expression of *ascl1b* and *NeuroD*, mediators of Notch signalling.

Reduced β-cell mass in *sept7b* knockdown larvae postulated that the larvae might not be able to produce enough insulin to maintain normal glucose levels, and indeed, we observed that *insa* expression was lower and whole body glucose level was increased in the absence of *sept7b*. Glucose administration stimulated *insa* expression in wild type and *sept7b* knockdown larvae as expected, but the level of *insa* remained very low in *sept7b*-depleted embryos. Insulin is the most important hormone that inhibits gluconeogenesis and similar to mammals, insulin suppresses the expression of the key gluconeogenic enzyme phosphoenolpyruvate carboxykinase 1 (*pck1*) in larval zebrafish^39^. Both wild type and *sept7b* knockdown larvae responded to glucose-stimulated insulin induction by reducing *pck1* expression, but *pck1* level in the knockdown larvae remained clearly higher compared to controls. This indicates defective glucoregulation in *sept7b*-depleted larvae.

In conclusion, the present study demonstrates that *sept7b*/septin 7 expression is conserved in the developing vertebrate pancreas, and that the multipotent pancreatic progenitors of the larval zebrafish do not reach the mature state upon knockdown of *sept7b*. The data indicate that *sept7b* is essential for the specification and differentiation of the endocrine cell types and that *sept7b* affects endocrine differentiation by modulating the activity of the Notch-pathway. Consequently, knockdown of *sept7b* disturbs glucose homeostasis. Further investigations will be required to define how septin 7 fits into the networks of other regulatory genes that play important roles in the endocrine cell differentiation and maintenance of glucose homeostasis. Delineation of these pathways will help to identify potential targets for pharmacological intervention to restore the β-cell mass in diabetes.

## Methods

### Zebrafish lines

The wild-type Turku zebrafish line, and *Tg(gcg:GFP), Tg(ptf1a:GFP)* and *Tg(NeuroD:GFP)* transgenic lines (kind gifts from Didier Stainier, Max Planck Institute for Heart and Lung Research, Germany) were maintained and raised as described previously^48^. Embryos were staged according to hours post fertilization (hpf) or days post fertilization (dpf). E3 medium containing 0.003% 1-phenyl 2-thiourea (PTU) to prevent pigmentation was used for larvae to be used for immunostaining or whole-mount *in situ* hybridization. The National Animal Experiment Board approved the protocols, and all animal experiments were performed according to approved guidelines.

### Immunostaining

For whole-mount immunostaining, zebrafish larvae at 3, 4 or 5 dpf were fixed in 4% paraformaldehyde (PFA) at 4 °C overnight followed by Dent’s fixative (80% methanol, 20% DMSO^49^) at −20 °C for 3–4 h. Samples were blocked with 5% normal goat serum (Sigma-Aldrich, St Louis, MO) in blocking solution (PBDT; PBS containing 1% bovine serum albumin), 1% DMSO, 0.2% Tween-20, 0.1% Triton X-100) and incubated with rabbit anti-septin 7 (C, Immuno-Biological Laboratories, Gumma, Japan or H-120, Santa Cruz Biotechnology, Dallas, TX), guinea pig anti-insulin (Dako Cytomation, Glostrup, Denmark) or guinea pig anti-Pdx1 (kind gift from Chris Wright, Vanderbilt University, TN, USA) in blocking solution at 4 °C overnight. Detection was performed with Alexa Fluor 488/555-conjugated donkey anti-rabbit or goat anti-guinea pig IgGs (Invitrogen). Images were acquired using a Leica SP8 confocal microscope with extended focus (Leica Microsystems CMS GmbH, Mannheim, Germany). Imaris v 7.6.3 software (BITPLANE Scientific Software, Zurich, Switzerland) was used to create 3D isosurface renderings from confocal z-stack images of β-cells, α-cells or whole zebrafish larvae. Insulin- or glucagon-positive cell volumes were segmented using the “background subtraction (local contrast)” thresholding option and the intensity threshold was set manually for each zebrafish larva. The volume of insulin- and glucagon-positive cell mass was measured relative to the total body volume of the zebrafish larva.

For histochemistry and immunohistochemistry, 4 or 5 dpf zebrafish larva were fixed in 4% PFA overnight, dehydrated in ethanol and embedded in paraffin. Longitudinal or transverse 4 μm sections were deparaffinised, rehydrated and stained with haematoxylin-eosin. Samples were photographed by using a Nikon Eclipse 800 microscope (Nikon Instruments Inc., Melville, NY, USA) equipped with Spot Image digital camera. For immunohistochemistry, slides were heated in citric acid, pH 6.0, washed with PBS and blocked with C-Block (Genemed Biotechnologies, South San Francisco, CA). Sections were incubated with rabbit anti-septin 7 (C or H-120) or guinea pig anti-insulin diluted in Dako REAL Antibody Diluent (Dako, Glostrup, Denmark) at 4 °C overnight, washed with PBST (PBS, 0.2% Tween 20) followed by incubation with Alexa Fluor 488/555-conjugated donkey anti-rabbit or goat anti-guinea pig IgGs (Invitrogen) at 4 °C for 2 h. Samples were imaged with Leica SP8 confocal microscope with extended focus or Zeiss Axioplan2 microscope (Carl Zeiss Microscopy GmbH, Jena, Germany).

### RT-PCR and Real-time qPCR

RNA was isolated from mouse tissues using the NucleoSpinRNAII kit (Macherey-Nagel, Düren, Germany) without on-column DNase treatment. DNase treatment was done separately followed by RNA purification with Nucleospin RNA CleanUp XS kit according to manufacturer’s instructions (Macherey-Nagel). Total RNA was reverse transcribed into cDNA with M-MLV reverse transcriptase (Promega, Madison, WI, USA) in reaction containing Oligo(dT)15 primers (Promega), random hexamers (Promega), the mix of all four dNTPs, and RNAse inhibitor (RiboLock; Fermentas, Thermo Fisher Scientific, Waltham, MA). Real-time PCR was done with SYBR Green JumpStart Taq ReadyMix for Quantitative PCR (Sigma-Aldrich, St Louis, MO) with a Corbett Rotor- Gene 6000 (Qiagen, Hilden, Germany). An exogenous positive control was used as a calibrator. Four dpf zebrafish larvae were exposed to 40 mM glucose (Sigma-Aldrich) for 24 h. Total RNA was isolated from 30 embryos using the RNeasy mini Kit (Qiagen, Hilden, Germany) and reverse transcribed with random hexamer primers and Superscript III reverse transcriptase (Invitrogen, Carlsbad, CA) following manufacturer’s protocol. qRT-PCR was performed using the Power SYBR Green PCR Master Mix (Applied Biosystems, Carlsbad, CA) in an iCycler iQTM Real-Time PCR Detection System (Bio-Rad Laborotories, Hercules, CA). Data were normalised to β-actin. The primers used are listed in Supplementary Table S1.

### Morpholino antisense oligonucleotide injections and rescue experiments

The morpholino antisense oligonucleotides (MO), *sept7b* TBMO 5′-TCGGGTCTCTCGATCATTGTCCTGT-3′ and a standard control MO 5′-CCTCTTACCTCAGTTACAATTTAT-3′, were obtained from Gene Tools (LLC, Philomath, OR). Validation of appropriate *sept7b* TBMO concentration and injection methods were described previously^15^. Rescue experiments were performed by co-injecting 250 pg full-length zebrafish *sept7b* capped mRNA with *sept7b* TBMO as described previously^15^.

### Cell labelling with EdU

EdU-staining was performed as previously described^26^. Four dpf zebrafish larvae were treated with 100 μM 5-ethynyl-2-deoxyuridine (EdU) for 24 h followed by fixation in 4% PFA. Embryos were washed in PBST and incubated in methanol at −20 °C for 3 h. EdU was detected using the Click-iT EdU Alexa Fluor 488 Imaging Kit (C10337; Invitrogen) according to manufacturer’s protocol. The larvae were rehydrated shortly through a methanol series into PBST and manually deyolked, incubated in Click-iT reaction cocktail at room temperature for 2 h followed by rinses in PBST. Following the EdU detection reaction, larvae were incubated in blocking buffer and immunostained with an antibody against insulin as described above.

### *In situ* hybridization

Whole mount *in situ* hybridization was performed as described^50^. The *trypsin* probe was a kind gift from Prof. Steven D. Leach (John Hopkins University, Baltimore USA) and the *shh* probes from Dr. Paola Bovolenta (Autonomous University of Madrid, Madrid, Spain). Image J software was used to define the length of the exocrine pancreas after *in situ* hybridization for *trypsin*.

### DAPT treatment

Stock solution of DAPT (N-[N-(3,5-Difluorophenacetyl)-L-alanyl]-S-phenylglycine t-butyl ester) (Sigma-Aldrich) was made in DMSO. Three dpf wild type of *sept7b* TBMO-injected larvae were treated with 20 μM or 100 μM DAPT in PTU-containing E3 medium for 48 h, replacing the DAPT after 24 h^26^. Control embryos were treated with 1% DMSO in PTU-containing E3 medium.

### Glucose assay

Absolute whole body glucose was measured from fifteen 5 dpf zebrafish larvae per condition, with at least 6 replicates, using a fluorescence-based enzymatic detection kit (Biovision Inc., California, USA) as previously described^51^. Detection was performed with a Fluoroskan Ascent FL Microplate Reader (Waltham, MA USA).

### Statistics

The significance of differences was analysed by using a two-tailed Student’s t-test. Error bars on the graphs show the standard error of the mean (SEM).

## Additional Information

**How to cite this article**: Dash, S. N. *et al. sept7b* is required for the differentiation of pancreatic endocrine progenitors. *Sci. Rep.* **6**, 24992; doi: 10.1038/srep24992 (2016).

## Supplementary Material

Supplementary Information
