# Supplementary material for: sept7b is required for the differentiation of pancreatic endocrine progenitors
Source: Sci Rep. 2016 Apr 26;6:24992. doi: 10.1038/srep24992 (PMC4845001; doi:10.1038/srep24992)
Supplement: Supplementary Information [file srep24992-s1.pdf]

sept7b is required for the differentiation of pancreatic endocrine progenitors

Surjya Narayan Dash[1], Elina Hakonen[2], Jarkko Ustinov[2], Timo Otonkoski[2], Olov Andersson[3], Sanna Lehtonen[1]

[1]Department of Pathology, University of Helsinki, Helsinki, Finland,
[2]Research Program for Molecular Neurology and Biomedicum Stem Cell Center, University of Helsinki, Helsinki, Finland,
[3]Department of Cell and Molecular Biology, Karolinska Institute, Stockholm, Sweden

Supplementary Figure S1. *sept7b* TBMO specifically knocks down *sept7b* in zebrafish larvae. (A-C) Images of zebrafish larvae injected with control MO **(A)**, *sept7b* TBMO **(B)** and *sept7b* TBMO together with *sept7b* cRNA **(C)** at 5 dpf. Knockdown of *sept7b* leads to pericardial (arrowhead) and yolk sac (arrow) edema, and co-injection of *sept7b* cRNA with *sept7b* TBMO rescues the phenotype. **(D)** The expression of septin 7 protein is reduced in *sept7b* TBMO-injected zebrafish larvae compared to control MO-injected larvae at 5 dpf. Larvae were lysed in RIPA buffer as previously described[1] and proteins were separated by SDS-PAGE. Western blotting was performed with a rabbit polyclonal antibody against septin 7 (Santa Cruz Biotechnology, Santa Cruz, CA) and a mouse monoclonal antibody against actin (Sigma-Aldrich, St Louis, MO) followed by Alexa-Fluor-680-conjugated donkey anti-rabbit (Invitrogen, Carlsbad, CA) and IR-Dye-800-conjugated donkey anti-mouse IgGs (LI-COR, Lincoln, NE). **(E)** qPCR reveals that the expression of *p21* mRNA shows a trend of downregulation in *sept7b* TBMO-injected larvae compared to control MO-injected larvae at 5 dpf. **(F)** Quantification of four replicate blots similar to the blot in **(A)** reveals significant downregulation of septin 7 protein in *sept7b* TBMO-injected larvae compared to control MO-injected larvae. Error bars represent mean ± SEM. ns, non-significant; * $p \leq 0.05$.

Supplementary Figure S2. **Pdx1-positive pancreatic cells are increased in *sept7b* knockdown larvae at 5 dpf. (A-D)** Pdx1-positive cells (red; arrows) in control MO-injected **(A-B)** and *sept7b* TBMO-injected **(C-D)** *Tg(ptf1a:GFP)* zebrafish larvae at 3

dpf. In **(A)** and **(C)** the exocrine pancreas is visualized by *ptf1a* (green) and the nuclei are labeled with DAPI (blue). **(B)** and **(D)** are corresponding images visualizing Pdx1 only. Asterisk (*) marks the exocrine pancreas. **(E)** Pdx1-positive cells are significantly increased in *sept7b* knockdown larvae compared to control MO-injected larvae. Error bars represent mean ± SEM. ** p≤ 0.005. Scale bar: A-D (25 μm).

Supplementary Figure S3. *NeuroD*-positive endocrine cells are increased in *sept7b* knockdown larvae. (A-B) *NeuroD*-positive cells (green) in control MO-injected **(A)** and *sept7b* TBMO-injected **(B)** *Tg(neuroD:GFP)* zebrafish larvae at 3 dpf. Nuclei are visualized with DAPI (blue). **(C)** *NeuroD*-positive cells are increased in *sept7b* knockdown larva compared to control MO-injected larvae. Error bars represent the standard error of mean. * p≤ 0.05. Scale bar: A, B (20 μm).

Supplementary Figure S4. Insulin-positive cells are increased in the intrapancreatic duct (IPD) of *sept7b* TBMO and DAPT -treated larvae. (A-D) Immunostaining of *Tg*(*ptf1a:GFP*) zebrafish larva co-treated with *sept7b* TBMO and 20 μM DAPT with antibodies against insulin shows cells positive for insulin (arrowheads) in the IPD **(B, D)** whereas wild type larva treated with 20 μM DAPT **(A, D)** does not. **(E)** Counting the number of cells positive for insulin revealed that 17 larvae depleted of *sept7b* and treated with 20 μM DAPT show altogether 12 cells positive for insulin in the IPD. In 17 wild type larvae treated with 20 μM DAPT altogether only four cells positive for insulin are observed in the IPD. In all cases, we observed only 0-2 cells/larva positive for insulin. Error bars represent the standard error of mean. * p≤ 0.05. Scale bar: A-D (25 μm).

References

1 Dash, S. N. *et al.* Sept7b is essential for pronephric function and development of left-right asymmetry in zebrafish embryogenesis. *J. Cell Sci.* **127**, 1476-1486 (2014).

Supplementary Figure S1

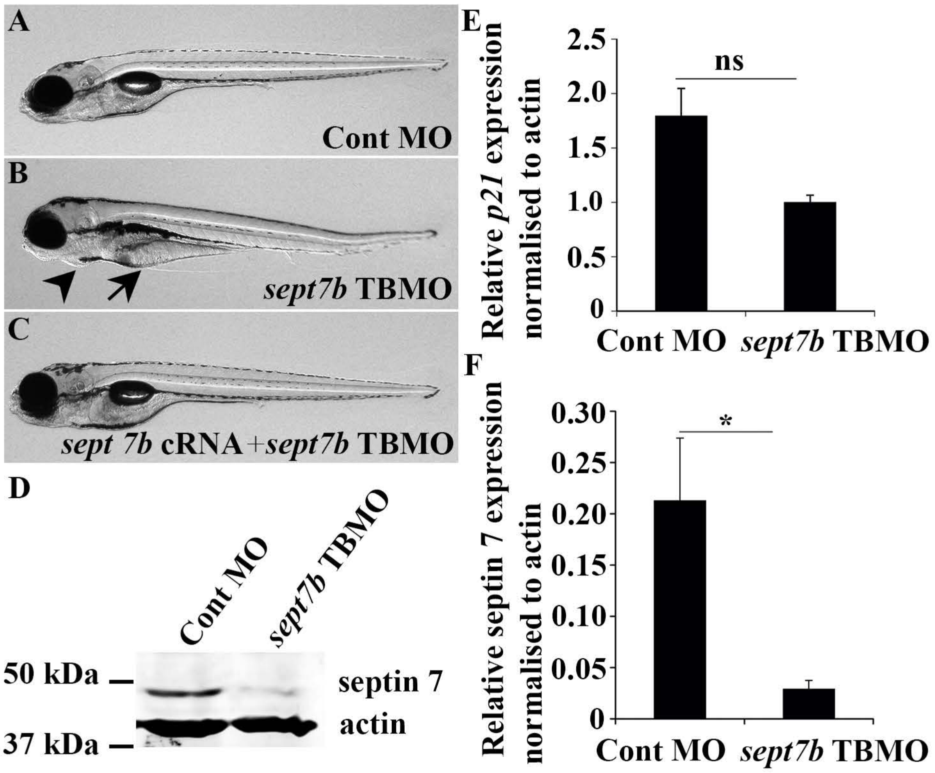

Supplementary Figure S2

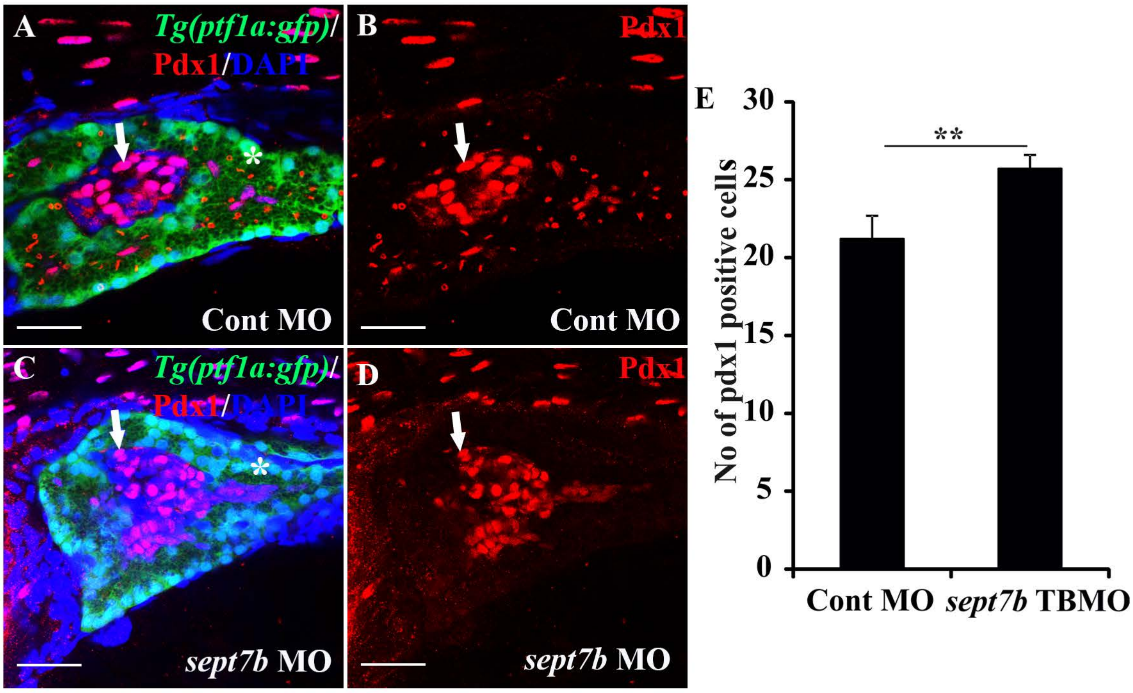

Supplementary Figure S3

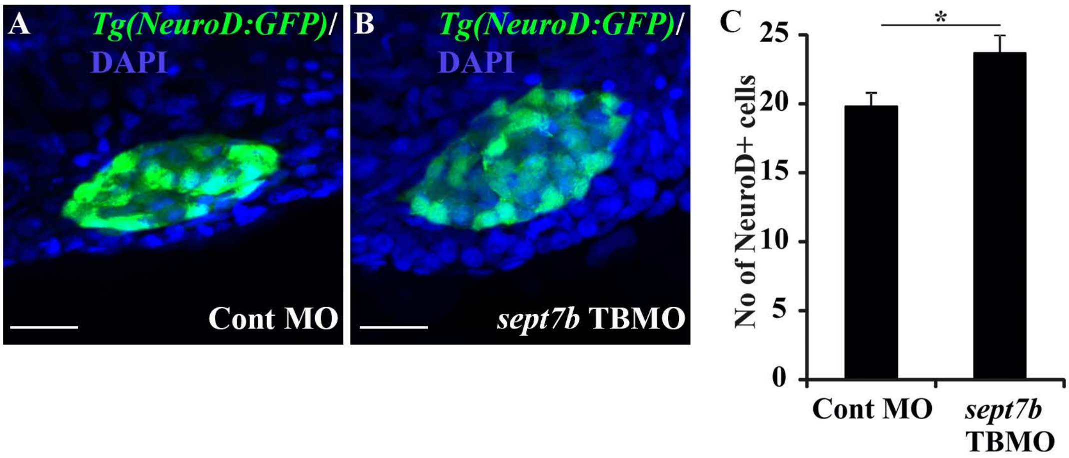

Supplementary Figure S4

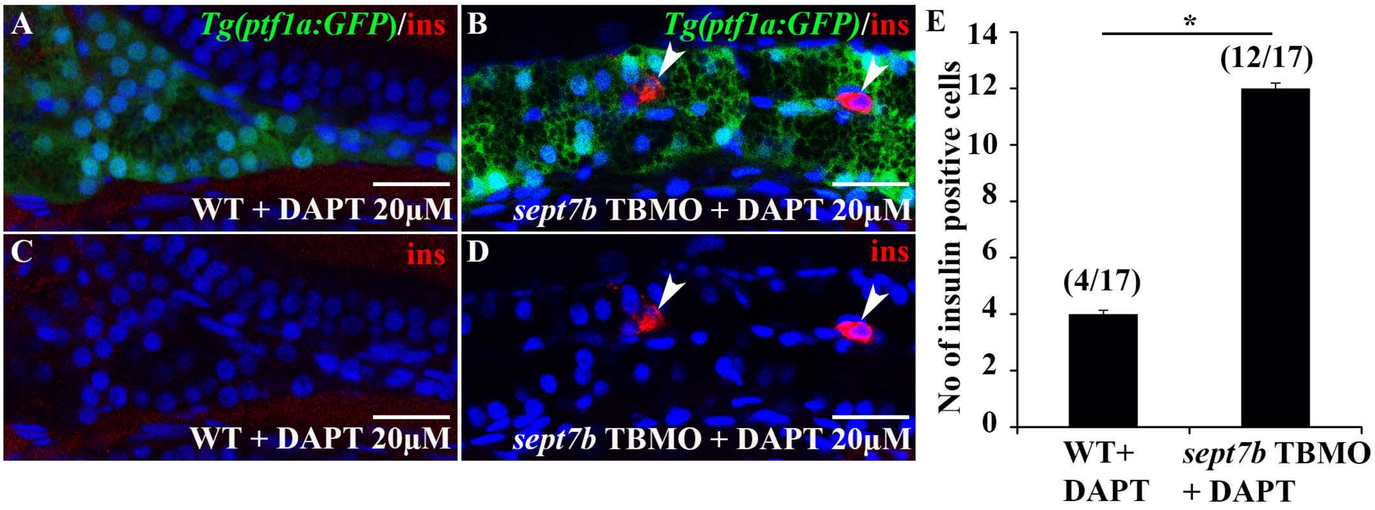

Supplementary Table S1

Primers used in the study.

sept7b zf-F	GACCATCTCAGAGGATCAAG
sept7b zf-R	ATGACAGGCTGCCAGCAGTT
insa zf-F	CTGTGTGGATCTCATCTGGT
insa zf-R	CTCTCTTCCTTATCAGCTCG
actin zf-F	CACTGGTTGTTGACAACGGA
actin zf-R	CATCACCAACGTAGCTGTCT
pck1 zf-F	TTCACTCAAGGCTCTCTCTC
pck1 zf-R	CACTGCTGTCGATGAACTCC
pdx1 zf-F	CAGTATACGCCTCACCATTG
pdx1 zf-R	CCGAGCGACTGTAGAGATGT
ptf1a zf-F	TGTGACGTTGGCAACTTCTC
ptf1a zf-R	CCTCCGCCTCTTCAGTAAGC
notch1a-F	CGACACCACACACACATGCT
notch1a-R	AGTGGCAGTTGTAGGTGTTG
notch1b-F	CAGTTATGAGTGCTCCTGTC
notch1b-R	GTTCACTCCATCCACACAGGTC
ascl1b-F	TTCAACGGACTGGGCTACAC
ascl1b-R	TCTGGAAGCCCATGTTGACC
p21-F (Robu et al., 2007)	CGGAATAAACGGTGTCGTCT
p21-R (Robu et al., 2007)	CGCAAACAGACCAACATCAC
Septin 7 mouse-F	AGAAGGTGGTGTTCAGTTGC
Septin 7 mouse-R	GACGTCTGTTCACTCGAGAT
GAPDH-mouse-F	GGTCATCCATGACAACTTTGG
GAPDH-mouse-R	CCATCCACAGTCTTCTGGGT
Cyclophilin G- mouse-F	CAATGGCCAACAGAGGGAAG
Cyclophilin G- mouse-R	CCAAAAACAACATGATGCCCA
